# Role of Delta-like 4 in Jagged1-induced tumour angiogenesis and tumour growth

**DOI:** 10.18632/oncotarget.16969

**Published:** 2017-04-08

**Authors:** Chern Ein Oon, Esther Bridges, Helen Sheldon, Richard C.A. Sainson, Adrian Jubb, Helen Turley, Russell Leek, Francesca Buffa, Adrian L. Harris, Ji-Liang Li

**Affiliations:** ^1^ Molecular Oncology Laboratories, Department of Oncology, Weatherall Institute of Molecular Medicine, University of Oxford, John Radcliffe Hospital, Oxford, UK; ^2^ Institute for Research in Molecular Medicine, Universiti Sains Malaysia, Penang, Malaysia; ^3^ Institute of Translational and Stratified Medicine, Plymouth University Peninsula Schools of Medicine and Dentistry, Plymouth, UK

**Keywords:** Notch signalling, DLL4, JAG1, angiogenesis, bevacizumab

## Abstract

Delta-like 4 (DLL4) and Jagged1 (JAG1) are two key Notch ligands implicated in tumour angiogenesis. They were shown to have opposite effects on mouse retinal and adult regenerative angiogenesis. In tumours, both ligands are upregulated but their relative effects and interactions in tumour biology, particularly in tumour response to therapeutic intervention are unclear. Here we demonstrate that DLL4 and JAG1 displayed equal potency in stimulating Notch target genes in HMEC-1 endothelial cells but had opposing effects on sprouting angiogenesis *in vitro*. Mouse DLL4 or JAG1 expressed in glioblastoma cells decreased tumour cell proliferation *in vitro* but promoted tumour growth *in vivo*. mDLL4-expressing tumours showed fewer but larger vessels whereas mJAG1-tumours produced more vessels. In both tumour types pericyte coverage was decreased but the vessels were more perfused. Both ligands increased tumour resistance towards anti-VEGF therapy but the resistance was higher in mDLL4-tumours versus mJAG1-tumours. However, their sensitivity to the therapy was restored by blocking Notch signalling with dibenzazepine. Importantly, anti-DLL4 antibody blocked the effect of JAG1 on tumour growth and increased vessel branching *in vivo*. The mechanism behind the differential responsiveness was due to a positive feedback loop for DLL4-Notch signalling, rendering DLL4 more dominant in activating Notch signalling in the tumour microenvironment. We concluded that DLL4 and JAG1 promote tumour growth by modulating tumour angiogenesis *via* different mechanisms. JAG1 is not antagonistic but utilises DLL4 in tumour angiogenesis. The results suggest that anti-JAG1 therapy should be explored in conjunction with anti-DLL4 treatment in developing anti-Notch therapies in clinics.

## INTRODUCTION

Angiogenesis is a multifaceted process involving matrix degradation, endothelial cell (EC) proliferation, migration, sprouting and recruitment of mural cells. Tumour angiogenesis is regulated by many angiogenic molecules including VEGF, which are produced by tumour or stromal cells within the tumour microenvironment [1]. Notch signalling can be activated in ECs following contact between stromal cells, ECs and tumour cells [2]. Both Delta-like 4 (DLL4) and Jagged1 (JAG1) ligands are implicated in tumour angiogenesis. DLL4 is predominantly expressed in the ECs of tumour blood vessels [3–10] but also in a small proportion of tumour cells [5, 7]; whereas JAG1, although also expressed in ECs [11, 12], is more highly expressed in tumour cells [13–16] and mural cells [17, 18]. These ligands have opposing effects on vessel formation. DLL4 has been shown to inhibit sprouting resulting in fewer but better perfused blood vessels, which promoted tumour growth [6–8, 19, 20]. JAG1 on the other hand can signal to both tumour cells and ECs [11, 12, 21] to promote angiogenesis and tumour growth *via* the MAPK pathway [16, 22]. Molecularly, DLL4 binds to the region of EGF-like repeats 1-13 on Notch1 while JAG1 binds to the repeats of 10-24 as the fragments containing such repeats effectively block the function of DLL4 and JAG1 respectively [23]. The 2.3-angstrom resolution crystal structure of the interacting regions of the Notch1-DLL4 complex further reveals a two-site, antiparallel binding orientation assisted by Notch1 O-linked glycosylation [24].

Interestingly, in a retina model the expression of DLL4 was shown to fluctuate dynamically in individual ECs within sprouting vessels [25] while JAG1 antagonised DLL4-Notch signalling during sprouting angiogenesis, thereby enhancing retina angiogenesis [26]. In the JAG1-engineered mice, EC-specific JAG1 deletion resulted in upregulation of DLL4-Notch signalling in the vessels shown by increasing HES1, HEY1 and DLL4 expression throughout ECs, reduced endothelial proliferation and decreased vessel branching [26]. Endothelial JAG1 also antagonized DLL4 regulation of endothelial branching and increased vascular maturation downstream of DLL4-Notch1 in a skin wound healing model [27] as well as promoted tumour growth through pro-angiogenic and angiocrine functions [28].

Many studies have focused on the effects of endothelial DLL4 or JAG1 on tumour growth and vascularisation. However, whether there is any function interaction between DLL4 and JAG and how such an interaction would affect tumour angiogenesis and tumour growth and progression are unknown. Prospectively, it would be important for targeted therapy if they were antagonistic in tumour. Therefore, we investigated different effects of DLL4 and JAG1 on *in vitro* angiogenesis, on xenograft tumour growth and vasculature, and on tumour response to anti-VEGF therapy.

## RESULTS

### DLL4 and JAG1 had opposite effects on sprouting angiogenesis *in vitro* by activating notch signalling

**Figure 1:**
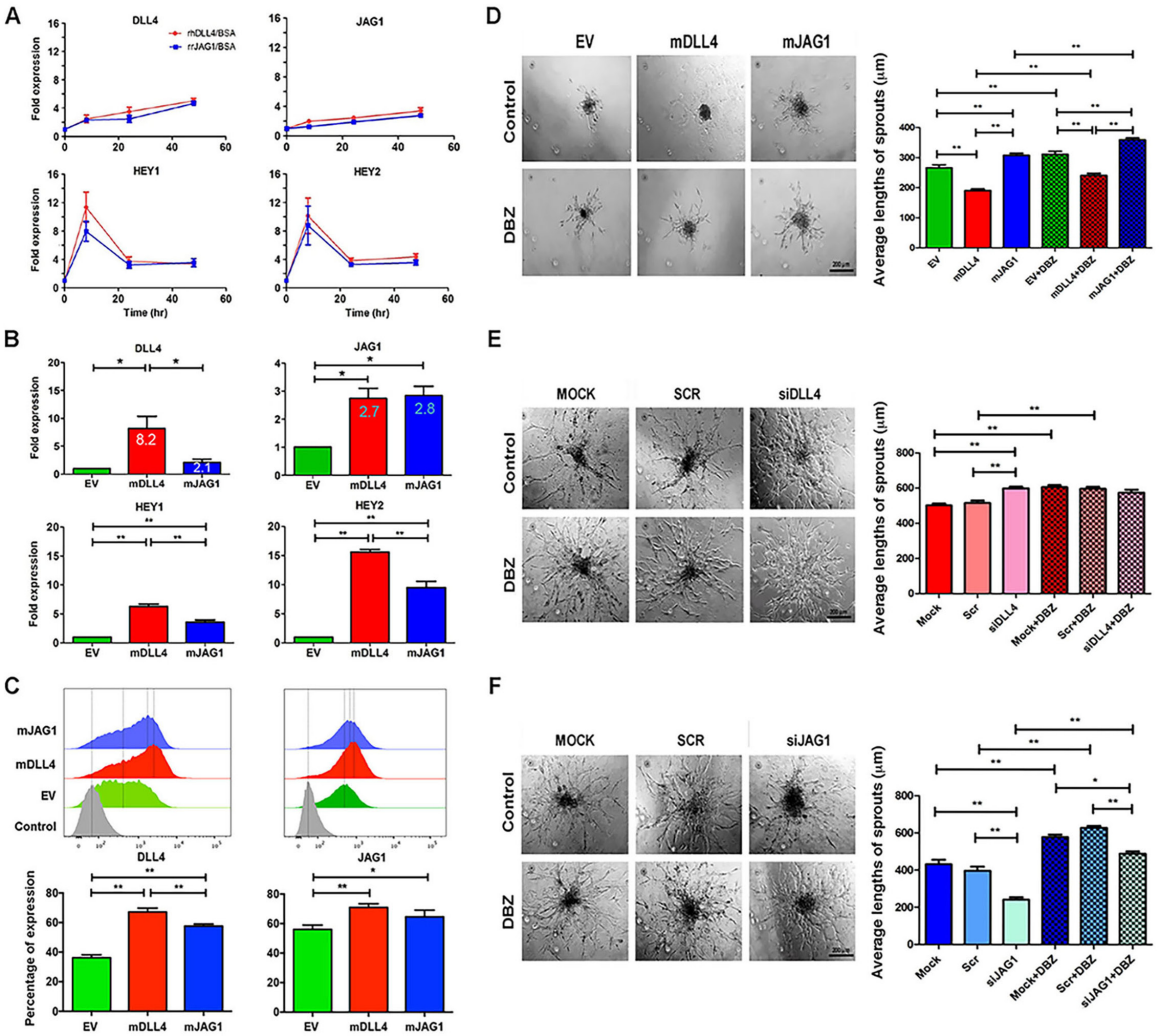
DLL4 and JAG1 activated Notch signalling and affected sprouting angiogenesis ***in vitro***. **A**. Expression profile of Notch target genes in HMEC-1 stimulated with rhDLL4 or rrJAG1 over a time course. QPCR was used to determine mRNA levels of DLL4, JAG1, HEY1 and HEY2. Fold changes were obtained by normalizing against the EV control.**B**. Expression profile of Notch target genes in HMEC-1 over-expressing mDLL4 or mJAG1 by retrovirus transductions. QPCR was used to determine mRNA levels of DLL4, JAG1, HEY1 and HEY2 (ANOVA with Bonferroni's post-test). **C**. Expression of endogenous hDLL4 and hJAG1 proteins in parental HMEC-1 cells (GFP-negative) sorted from co-culture of EV-, mDLL4- or mJAG1-overexpressing HMEC-1 with an equal amount of parental HMEC-1 cells by FACS analysis (ANOVA with Bonferroni's post-test). **D**. Effect of mDLL4 and mJAG1 expressed in HMEC-1 cells on sprouting in HMEC-1 spheroids and treated with DBZ. Average lengths of three longest sprouts were calculated for statistical test (ANOVA with Bonferroni's post-test). Graphs are means of 3 independent experiments. **E**. Effect of knockdown of DLL4 in HMEC-1 cells by specific siDLL4 on sprouting in HMEC-1 spheroids. Average lengths of three longest sprouts were calculated for statistical test (ANOVA with Bonferroni's post-test). Graphs are means of 3 independent experiments. **F**. Effect of knockdown of JAG1 in HMEC-1 cells by specific siJAG1 on sprouting in HMEC-1 spheroids. Average lengths of three longest sprouts were calculated for statistical test (ANOVA with Bonferroni's post-test). Graphs are means of 3 independent experiments. *P<0.05, ** *P*<0.01.

Both rhDLL4 and rrJAG1, when coated on plates (18nM, chosen from dose response curves to induce target genes maximally, data not shown), were capable of upregulating the expression of DLL4, JAG1, HEY1, and HEY2 in HMEC-1 at mRNA levels with an equal potency (Figure 1A). However, rhDLL4 upregulated DLL4, JAG1, HEY1 and HEY2 more strongly in HUVECs than rrJAG1 did (Supplementary Figure S1A).

DLL4 and JAG1 were then upregulated by retroviral transductions (Supplementary Figure S1B) and downregulated by knockdown of endogenous hDLL4 and hJAG1 in HMEC-1 respectively (Supplementary Figure S1C and S1D). When stably expressed in HMEC-1, mDLL4 increased the expression of endogenous hDLL4 approximately 8.2-fold whereas mJAG1 only increased hDLL4 expression about 2.1-fold (Figure 1B). Induction of HEY1 and HEY2 was also higher in mDLL4-expressing cells compared to mJAG1-expressing cells. However, both mDLL4 and mJAG1 equally upregulated the expression of endogenous JAG1 (2.7-fold *versus* 2.8-fold) (Figure 1B).

FACS analysis for measuring the expression of hDLL4 or hJAG1 at protein levels in parental HMEC-1 cells that were sorted from co-culture of ligand-overexpressing HMEC-1 cells by mixing an equal amount of parental cells showed that mDLL4 stimulated the expression of endogenous hDLL4 significant stronger than mJAG1 did, whereas both mDLL4 and mJAG1 equally induced the expression of endogenous hJAG1 compared to the EV control (Figure 1C), consistent with the mRNA results.

The role of these ligands in endothelial sprouting was investigated using hanging drop assays. mDLL4 decreased sprouting while mJAG1 promoted sprouting of HMEC-1 (Figure 1D). Blocking Notch signalling with DBZ increased sprouting in all EV-, mDLL4- and mJAG1-spheroids. Knockdown of endogenous hDLL4 (Figure 1E) or hJAG1 (Figure 1F) by specific siRNAs showed opposite effects on sprouting angiogenesis. siDLL4 significantly increased sprouting (Figure 1E) while siJAG1 decreased sprouting (Figure 1F). Treatment with DBZ increased sprouting in controls and abolished the siJAG1 suppressive effect by increasing sprouting.

### DLL4 and JAG1 reduced cell proliferation *in vitro* but promoted tumour growth *in vivo* through different vasculature phenotypes

**Figure 2:**
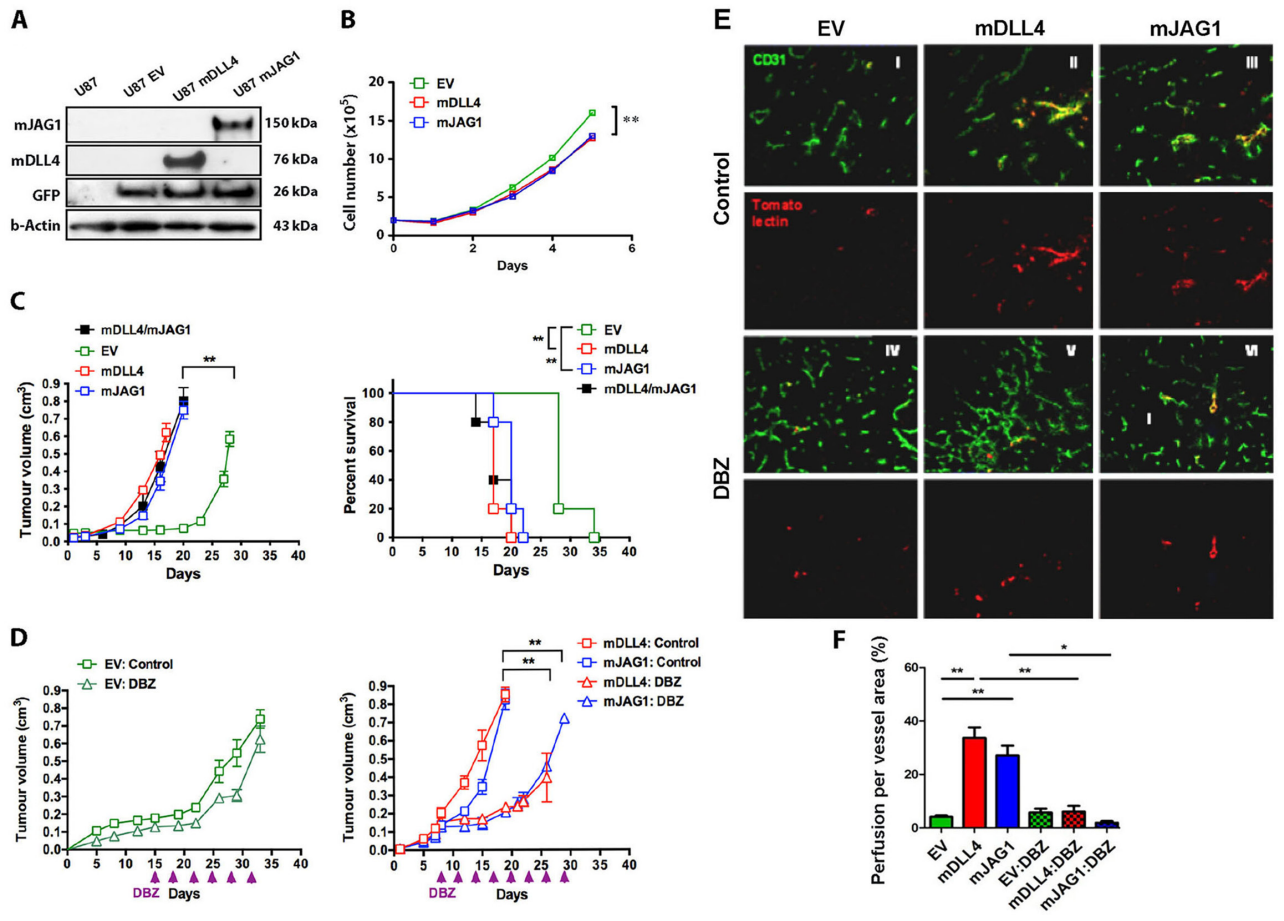
mDLL4 and mJAG1 reduced proliferation ***in vitro*** but promoted tumour growth of U87 cells ***in vivovia*** different vasculature. **A**. Upregulation of mDLL4 and mJAG1 in U87 cells by retrovirus transductions. Western blotting confirmed the expression of mDLL4 and mJAG1. GFP encoded by the retrovirus vector was served as an internal control for the transduction efficacy. β-actin was served as a protein loading control. **B**. *In vitro* cell proliferation assays of U87 cells over a time course. N = 4. Error bars represent SD. **C**. Tumour growth and mouse survival of U87-EV, U87-mDLL4, U87-mJAG1 and U87-mDLL4+U87-mJAG1 (50%/50%) in mice. Parametric generalized linear model with random effects was used for tumour growth test and Kaplan-Meier analysis for overall survival test. Each group consisted of 5 mice. **D**. Tumour growth of U87-EV, U87-mDLL4 and U87-mJAG1 in mice treated with DBZ (8.1µmol/kg body weight, ip every 3 days). Each group consisted of 5 mice. Parametric generalized linear model with random effects was used for statistical test. **E**. Tumour vascular phenotype and vascular perfusion results. Immunofluorescence double staining for vessels (CD31, green) and tomato-lectin (red). Magnification 200X. **F**. Quantification of vascular perfusion. Graphs are means of perfusion per vessel area in 5 tumours. ANOVA with Bonferroni's post-test. *P<0.05, ** *P*<0.01, *** *P*<0.001.

mDLL4 or mJAG1, expressed in U87 cells as confirmed by Western blotting (Figure 2A), reduced cell proliferation *in vitro* (Figure 2B) but promoted tumour growth *in vivo* and decreased mouse survival (Figure 2C). Implantation of an equal amount of mDLL4- and mJAG1-expressing tumour cells in mice produced similar results in terms of tumour growth and mouse survival to that of mDLL4- or mJAG1-expressing tumour (Figure 2C). DBZ did not seem to significantly affect xenograft growth of EV cells but abolished the promotion of tumour growth by either mDLL4 or mJAG1 (Figure 2D), suggesting that promotion of tumour growth by both ligands were mediated by activation of Notch signalling.

Immunofluorescent staining of tumour sections with anti-CD31 antibody showed that mDLL4 expressed in tumour cells reduced vessel density but increased vessel size compared to the EV control (Figure 2E), consistent with the result obtained from hDLL4 [6]. In stark contrast, mJAG1 expressed in tumour cells dramatically increased vessel density but not vessel size. DBZ treatment not only increased vessel density in all three types of tumours but also abolished the larger vessels in mDLL4-tumour, suggesting that the larger vessels induced by mDLL4 were promoted by mDLL4-Notch signalling. Vascular perfusion assays by a tail vein injection of tomato-lection into the tumour and staining on tumour sections demonstrated that both mDLL4 and mJAG1 significantly increased vascular perfusion in mDLL4- and mJAG1-tumour compared to that of EV-tumour (Figure 2E and 2F). DBZ treatment did not significantly affect vascular perfusion in EV-tumour but abolished the promotion of vascular perfusion in either mDLL4- or mJAG1-tumour.

mDLL4 or mJAG1 expressed in other three tumour cell lines as confirmed by FACS analysis (Supplementary Figure S2A) significantly reduced *in vitro* growth of SAS cells but not PC3 and DU145 cells (Supplementary Figure S2B). However, there were no significant effects on either tumour growth or mouse survival for all these three tumour types compared to the relevant EV-control (Supplementary Figure S2C). Tumour vasculature evaluation revealed that mDLL4 significantly decreased vessel number but increased vessel size compared to the EV control while mJAG1 greatly increased vessel number but not vessel size in all these three tumour types (Supplementary Figure S2D).

### DLL4 and JAG1 mediated tumour resistance to anti-VEGF therapy with bevacizumab

**Figure 3:**
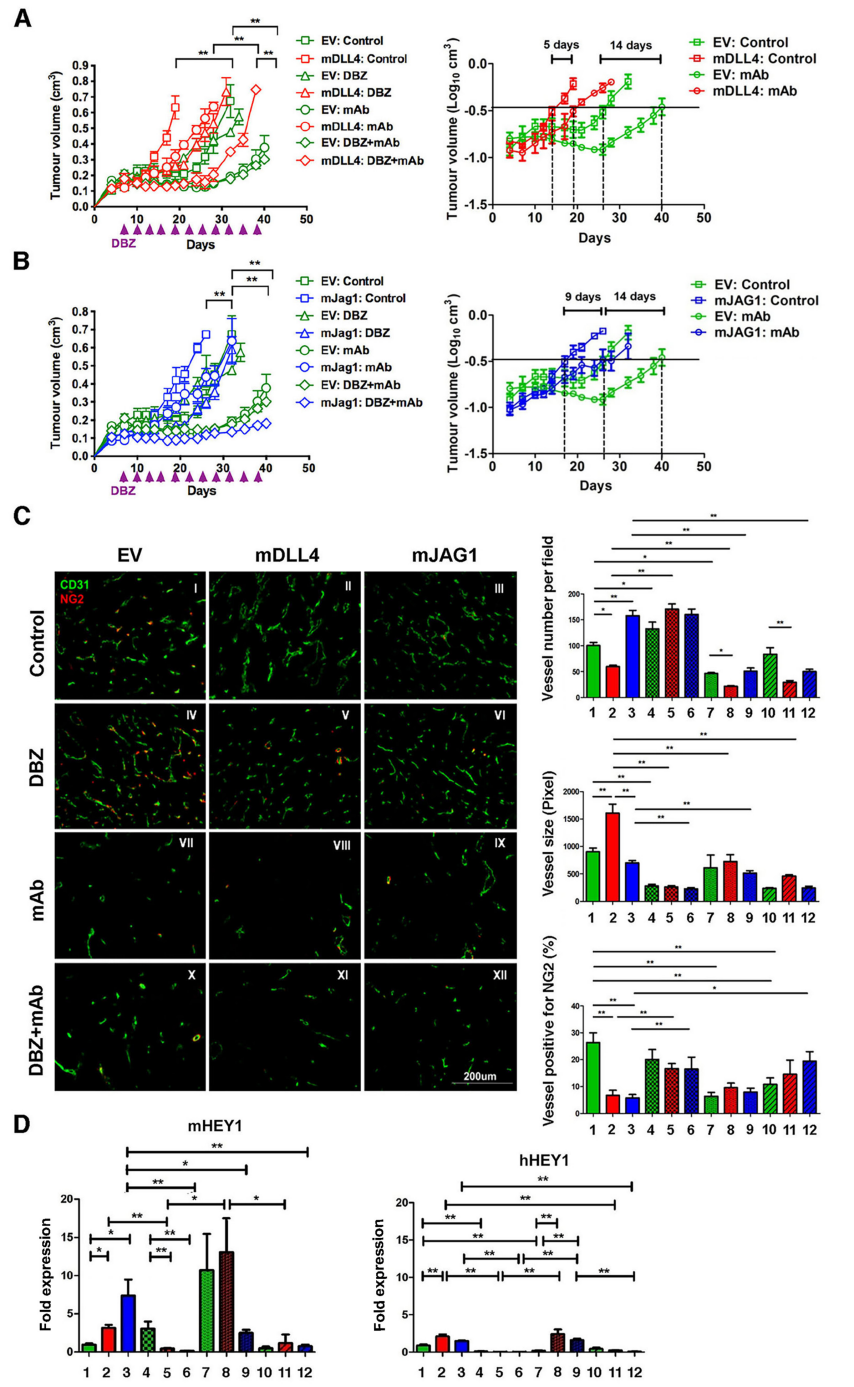
DLL4 and JAG1 mediated tumour resistance to anti-VEGF therapy **A**. Tumour growth curves (left panel) and linear plot of tumour volume (right panel) of U87-EV and U87-mDLL4 treated with PBS control, DBZ, bevacizumab (mAb), and combination of DBZ with bevacizumab. Each group consisted of 5 mice. A generalized linear model with random effects for tumour growth was used to analysis growth curves. ANOVA F-test was used to assess significance between curve fits. **B**. Tumour growth curves (left panel) and linear plot of tumour volume (right panel) of U87-EV and U87-mJAG1 treated with PBS control, DBZ, bevacizumab (mAb), and combination of DBZ with bevacizumab. Each group consisted of 5 mice. **C**. Tumour vascular phenotypes revealed by immunofluorescence double staining for vessels (CD31, green) and for pericytes (NG2, red) (left panel) as well as quantifications of vessel number, vessel size and vessel positive for pericytes (right panels). Immunofluorescent images (magnification 200X) were acquired for five randomly chosen fields per tumour section. Each group consisted of 5 tumours. ANOVA with Bonferroni's post-test.**D**. Quantification of Notch target expression of mHEY1 in tumour stroma (mouse) and hHEY in tumour cells (human) by QPCR. ANOVA with Bonferroni's post-test. Each group consisted of 5 tumours. *P<0.05, ** *P*<0.01. Each image in left panel or column in right panels represents: I/1, U87-EV control; II/2, U87-mDLL4 control; III/3, U87-mJAG1 control; IV/4, U87-EV DBZ; V/5, U87-mDLL4 DBZ; VI/6, U87-mJAG1 DBZ; VII/7, U87-EV mAb; VIII/8, U87-mDLL4 mAb; IX/9, U87-mJAG1 mAb; X/10, U87-EV DBZ+mAb; XI/11, U87-mDLL4 DBZ+mAb; XII/12, U87-mJAG1 DBZ+mAb.

Bevacizumab treatment of EV-tumour significantly delayed tumour growth (Figure 3A) compared to vehicle control (EV:mAb *versus* EV:Control). mDLL4 expressed in U87 cells conferred tumours more resistance to anti-VEGF therapy as shown by the shorter delay for 5 days (mDLL4:control versus mDLL4:mAb) compared to the 14-day delay in EV-tumours (EV:control *versus* EV:mAb). Similarly, mJAG1 expressed in U87 also instigated tumour resistance to anti-VEGF therapy (Figure 3B) as reflected by the growth delay for 9 days (mJAG1:control *versus* mJAG1:mAb) compared to the 14-day delay in EV-tumours (EV:control *versus* EV:mAb). However, such resistance contributed by mJAG1 (5-day difference, Figure 3B) was significantly weaker than that contributed by mDLL4 (9-day difference, Figure 3A).

DBZ treatment did not significantly affect EV-tumour growth compared to vehicle control (EV:DBZ *versus* EV:Control) but dramatically postponed xenograft growth of either mDLL4-tumour (mDLL4:DBZ *versus* mDLL4:Control, Figure 3A) or mJAG1-tumour (mJAG1:DBZ *versus* mJAG:Control, Figure 3B).

Combined DBZ and bevacizumab treatment exhibited an additive inhibitory effect on the growth of mDLL4-tumours when compared to either DBZ (mDLL4:DBZ+mAb *versus* mDLL4:DBZ) or bevacizumab therapy (mDLL4:DBZ+mAb *versus* mDLL4:mAb) (Figure 3A) but such an effect was not clear for EV-tumours (EV:DBZ+mAb *versus* EV:mAb). Combined DBZ with bevacizumab treatment (mJAG1:DBZ+mAb) showed a synergistic inhibition effect on the growth of mJAG1-tumours compared to DBZ (mJAG1:DBZ) or bevacizumab (mJAG1:mAb) alone (Figure 3B).

### Therapeutic effects on tumour vasculature

Expression of mDLL4 decreased vessel number but increased vessel size, whereas expression of mJAG1 increased vessel number but not vessel size when compared to EV control. Pericyte coverage, as detected by NG2 staining [29], decreased significantly in both mDLL4- and mJAG1-tumours when compared to that in EV-tumour (II-III *versus* I) (Figure 3C).

Bevacizumab reduced vessel density in EV-, mDLL4- and mJAG1-tumours (VII-IX *versus* I-III) but did not affect pericyte coverage in mDLL4- and mJAG1-tumours (VIII-IX *versus* II-III).

DBZ treatment increased vessel numbers in EV and mDLL4 groups (IV-V *versus* I-II) but significantly decreased vessel size in EV, mDLL4 and mJAG1 groups (IV-VI *versus* I-III). Pericyte coverage was also significantly increased in DBZ-treated mDLL4 and mJAG1 groups (V-VI *versus* II-III).

The percentage of pericyte coverage in over-expressing tumours treated with both DBZ and bevacizumab was similar to that in tumours treated with DBZ alone (XI-XII *versus* V-VI).

Notch activation in all these tumours was confirmed by verifying the expression of hHEY1 from human tumour cells and mHEY1 from mouse stromal cells with QPCR (Figure 3D). Higher expression of mHEY1 was detected in all tumours compared to hHEY1 expression. DBZ inhibited Notch signalling in tumours as shown by down-regulation of both mHEY1 and hHEY1 in mDLL4- and mJAG-tumours.

### DLL4 and JAG1 induced endogenous DLL4 expression in tumour tissues and vessels

**Figure 4:**
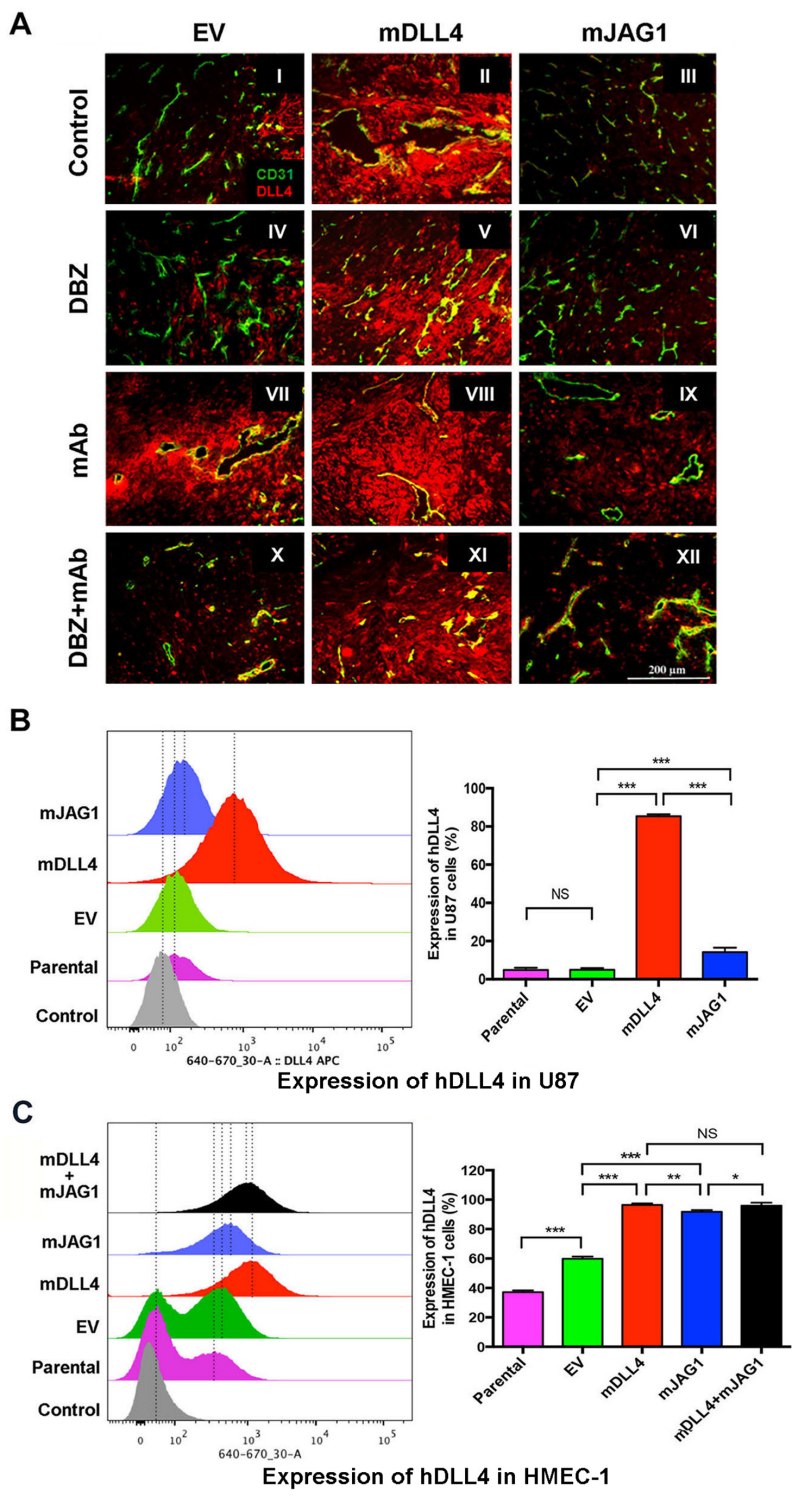
DLL4 and JAG1 induced endogenous DLL4 expression in tumour vessels **A**. Expression of DLL4 (red) and CD31 (green) as revealed by immunofluorescence double staining of tumour sections of U87. Magnification 200X.**B**. Endogenous hDLL4 expression in parental U87 cells (GFP-negative) sorted from co-culture of parental U87, U87-EV, U87-mDLL4 or U87-mJAG1 (GFP-positive) with an equal amount of parental U87 cells. hDLL4 expression in GFP-negative U87 cells was detected by FACS staining with anti-DLL4 antibody (recognised both human and mouse DLL4). ANOVA with Bonferroni's post-test. NS, no statistical difference. N = 3. Error bars represent SD. **C**. Endogenous hDLL4 expression in parental HMEC-1 cells (GFP-negative) sorted from co-culture of parental U87, U87-EV, U87-mDLL4 or U87-mJAG1 (GFP-positive) with an equal amount of parental HMEC-1 cells. hDLL4 expression in GFP-negative HMEC-1 cells was detected by FACS staining with anti-DLL4 antibody. ANOVA with Bonferroni's post-test. NS, no statistical difference. N = 3, Error bars represent SD. *P<0.05, ** *P*<0.01, *** *P*<0.001.

Endogenous DLL4 was abundantly expressed in tumour vasculature (Figure 4A I-III) as revealed by anti-mouse and human DLL4 antibody staining. DLL4 expression was also very high in mDLL4-tumours, confirming the over-expression in these cells, but also in the vessels (II, V, VIII and XI), suggesting a positive feedback loop. Expression of endogenous DLL4 in the vessels was diminished with DBZ treatment in EV-tumours and mJAG1-tumours (IV and VI). However the reduction of DLL4 expression in vessels was not prominent in mDLL4-tumours treated with DBZ, bevacizumab or combination (V, VIII and XI), suggesting the enhanced expression of endogenous DLL4 by mDLL4 was more resistant to reversal by DBZ.

Bevacizumab profoundly pruned the vessels leaving behind surviving vessels that expressed high levels of DLL4 in all three types of tumours (VII-IX). Increased DLL4 expression was observed in EV-tumours treated with bevacizumab compared to control (VII *versus* I) as DLL4 is induced by hypoxia through HIF-1α [10]. Lesser DLL4 expression was observed in the vasculature of mJAG1-tumours compared to mDLL4-tumours when treated with bevacizumab (IX *versus* VIII) as well as treated with the combination of DBZ and bevacizumab (XII *versus* XI) but more DLL4 expression occurred in the vasculature of mJAG1-tumour than in EV-tumours when treated with the combination (XII *versus* X). DLL4 stained positively on tumour and/or stromal cells, and increased especially in cells surrounding tumour vessels when EV-tumours and mJAG1-tumours were treated with bevacizumab (VII and IX) as well as treated with the combination (X and XII).

To validate the *in vivo* results, we performed *in vitro* co-culture assays by mixing of an equal amount of over-expressing cells containing GFP expression encoded by the retrovirus vector with parental cells without retrovirus transduction (GFP-negative), and detected endogenous expression of hDLL4 or hJAG1 in parental (neighbouring) cells by FACS staining with anti-DLL4 or JAG1 mAbs respectively (Supplementary Figure S3). mDLL4 expressed in U87 cells (Figure 4B) greatly stimulated endogenous hDLL4 expression in neighbouring U87 cells compared to either parental basal or EV control (mDLL4 *versus* EV or parental). mJAG1 expressed in U87 cells also significantly stimulated endogenous hDLL4 expression in neighbouring U87 cells compared to both controls (mJAG1 *versus* EV or parental) but substantially weaker than mDLL4 did (mJAG1 *versus* mDLL4). Co-culture of mDLL4-expesssing U87 with HMEC-1 cells (Figure 4C) demonstrated that mDLL4 expressed in U87 significantly induced hDLL4 expression in HMEC-1 cells compared to EV control (mDLL4 *versus* EV). Similarly, mJAG1 expressed in U87 cells greatly induced hDLL4 expression in HMEC-1 cells compared to EV control (mJAG1 *versus* EV) but significantly weaker than mDLL4 did (mJAG1 *versus* mDLL4). Mixture of mDLL4-expressing with mJAG1-expressing U87 cells induced hDLL4 expression in HMEC-1 as did by mDLL4-expressing U87 alone (mDLL4+mJAG1 *versus* mDLL4).

Similar experiments were also performed for SAS, PC3 and DU145 tumour cells (Supplementary Figure S4). *In vivo*, both mDLL4 and mJAG1 increased the expression of DLL4 in tumour tissues but there was more induction by mDLL4 than by mJAG1 (Supplementary Figure S4A). *In vitro*, similar results were obtained from the co-culture assays of SAS (Supplementary Figure S4B), PC3 (Supplementary Figure S4C) or DU145 cells (Supplementary Figure S4D).

### DLL4 and JAG1 increased endogenous JAG1 expression in tumour tissues

**Figure 5:**
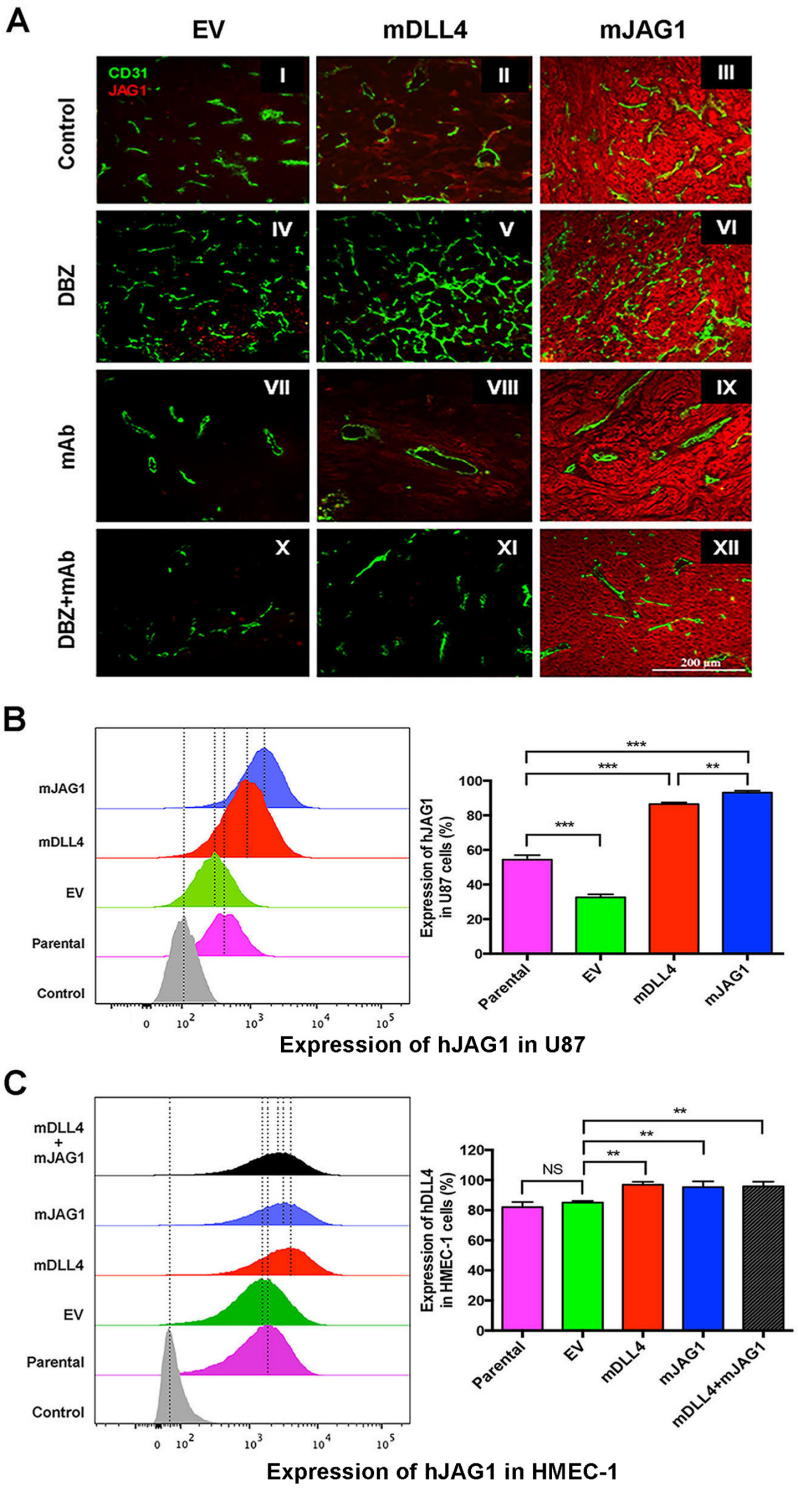
DLL4 and JAG1 induced endogenous JAG1 expression in tumour tissues **A**. Expression of JAG1 (red) and CD31 (green) as revealed by immunofluorescence double staining of tumour sections of U87. Magnification 200X. **B**. Endogenous hJAG1 expression in parental U87 cells (GFP-negative) sorted from co-culture of parental U87, U87-EV, U87-mDLL4 or U87-mJAG1 (GFP-positive) with an equal amount of parental U87 cells. hJAG1 expression in GFP-negative U87 cells was detected by FACS staining with anti-human JAG1 mAb (64D). ANOVA with Bonferroni's post-test. N = 3, Error bars represent SD. **C**. Endogenous hJAG1 expression in parental HMEC-1 cells (GFP-negative) sorted from co-culture of parental U87, U87-EV, U87-mDLL4 or U87-mJAG1 (GFP-positive) with an equal amount of parental HMEC-1 cells. hJAG1 expression in GFP-negative HMEC-1 cells was detected by FACS staining with anti-human JAG1 mAb (64D). ANOVA with Bonferroni's post-test. NS, no statistical difference. N = 3, Error bars represent SD. *P<0.05, ** *P*<0.01, *** *P*<0.001.

Endogenous JAG1 was weakly expressed in the vessels (Figure 5A). Notably, expression of endogenous JAG1 intensified in tumour and stromal cells of mDLL4-tumour compared to that of EV-tumour (II *versus* I). JAG1 was abundantly expressed in mJAG1-expressing tumours ratifying the transduction efficacy (III, VI, IX and XII). Inhibition with DBZ resulted in abated JAG1 expression in mDLL4-tumours compared to that of mDLL4-tumour control (V *versus* II) while bevacizumab had no significant effects on JAG1 expression in all three types of tumours compared to that of all control tumours (VII-IX *versus* I-III). Combined treatment with DBZ and bevacizumab dramatically reduced expression of endogenous JAG1 in both EV- and mDLL4-tumours (X-XI *versus* I-II).

*In vitro* co-culture experiments showed that either mDLL4 or mJAG1 expressed in U87 cells dramatically induced endogenous hJAG1 expression in neighbouring U87 cells (Figure 5B) although the intensity was slightly higher induced by mJAG1 than by mDLL4 in U87 cell (mJAG1 *versus* mDLL4). Co-culture of over-expressing U87 cells with HMEC-1 cells revealed that either mDLL4 or mJAG1 expressed in U87 cells significantly stimulated endogenous hJAG1 expression in HMEC-1 cells compared to the EV control (Figure 5C).

Similar experiments were performed for SAS, PC3 and DU145 tumour cells (Supplementary Figure S5). *In vivo*, both mDLL4 and mJAG1 expressed in tumour cells increased endogenous JAG1 expression in tumour tissues of either SAS (Supplementary Figure S5A) or PC3 (Supplementary Figure S5B). *In vitro*, similar results were obtained from the co-culture assays of SAS (Supplementary Figure S5C) and DU145 (Supplementary Figure S5D) but the intensity of endogenous hJAG1 induced by mJAG1 was higher than by mDLL4.

### DLL4 rather than JAG1 decreased tumour hypoxia

**Figure 6:**
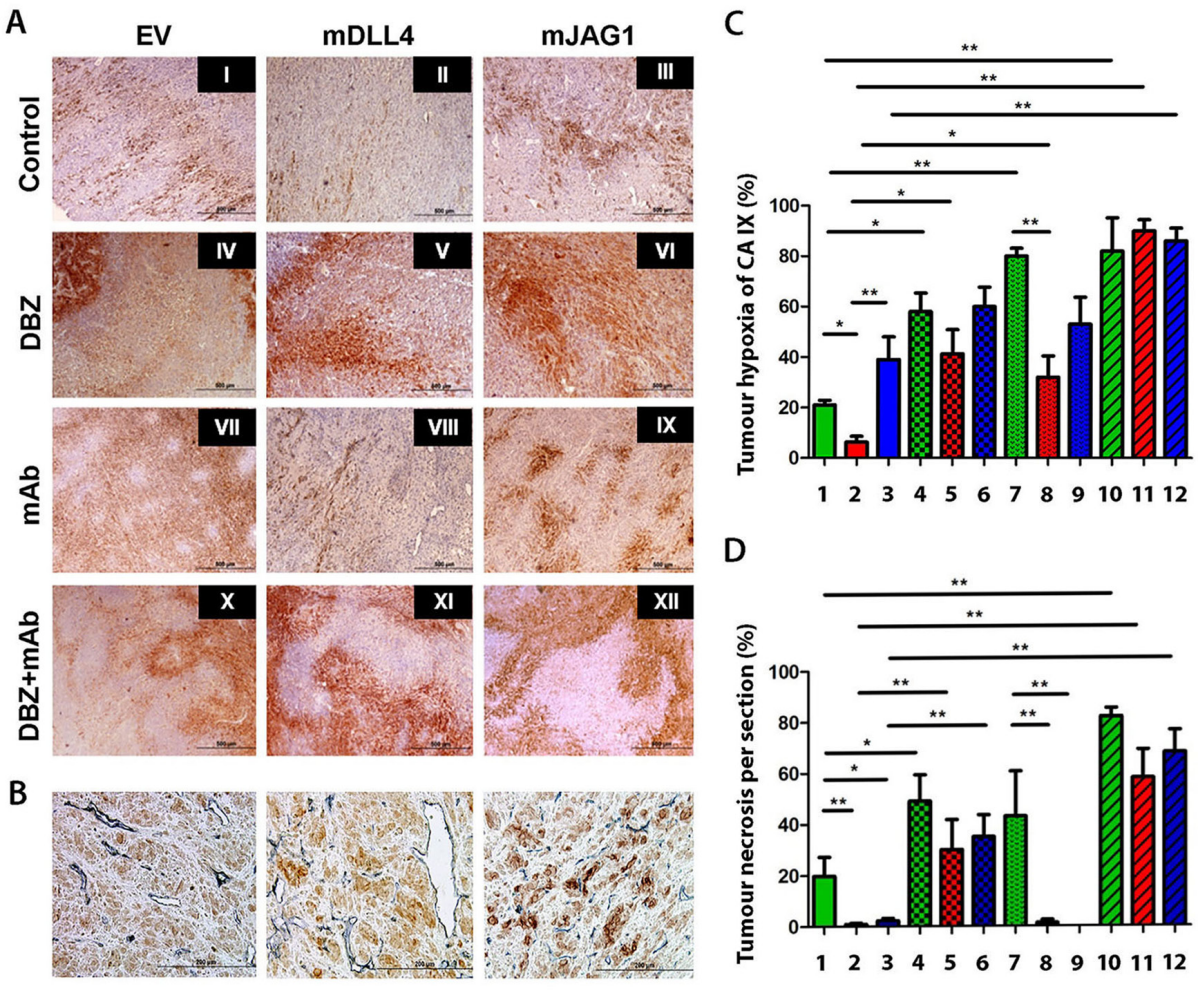
Immunohistochemical staining for hypoxia and tumour vessels in xenograft tumours **A**. Intra-tumour hypoxia staining of U87 tumour sections using carbonic anhydrase IX (CAIX) (brown). **B**. Double staining for CAIX (brown) and blood vessels (CD34) (blue) on tumour sections of U87. **C**. Tumour hypoxia quantification. ANOVA with Bonferroni's post-test. Each group contained 5 tumours. **D**. Tumour necrosis quantification. ANOVA with Bonferroni's post-test. Each group contained 5 tumours. *P<0.05, ** *P*<0.01. Each image in left panel or column in right panels represents: I/1, U87-EV control; II/2, U87-mDLL4 control; III/3, U87-mJAG1 control; IV/4, U87-EV DBZ; V/5, U87-mDLL4 DBZ; VI/6, U87-mJAG1 DBZ; VII/7, U87-EV mAb; VIII/8, U87-mDLL4 mAb; IX/9, U87-mJAG1 mAb; X/10, U87-EV DBZ+mAb; XI/11, U87-mDLL4 DBZ+mAb; XII/12, U87-mJAG1 DBZ+mAb.

Consistent with our previous work with hDLL4 [7], mDLL4 significantly diminished CAIX expression in mDLL4-tumours compared to that in EV-tumours (Figure 6) as revealed by CAIX (brown) (Figure 6A) and CD34 (grey) dual staining (Figure 6B) and by its quantification (Figure 6C). A weaker CAIX staining was observed around bigger and perfused vessels in mDLL4-tumours (Figure 6B). This effect was reversed when Notch signalling was blocked with DBZ treatment rendering the tumours more hypoxic in EV and mDLL4 groups (IV-V *versus* I-II). Both groups had an increase in hypoxia with either DBZ (IV-V) or bevacizumab treatment (VII-VIII) with an additive effect with combination treatment seen in mDLL4-tumours (XI). In contrast, expression of mJAG1 did not significantly reduce CAIX expression compared to EV-tumours (III *versus* I). Treatment of mJAG1-tumours with DBZ (VI) or bevacizumab (IX) did not significantly affect the level of CAIX expression compared to control-treated mJAG1 tumours (III) but the combination treatment significantly increased hypoxia (XII *versus* III).

Both mDLL4 and mJAG1 significantly reduced tumour necrosis in either mDLL4- or mJAG1-tumours (Figure 6A and 6D) compared to that of EV-tumours (II-III *versus* I) while treatment with DBZ obviated the effect and increased necrosis (IV-VI *versus* I-III). Bevacizumab treatment increased necrosis in EV-tumours (VII *versus* I) but not in mDLL4- and mJAG1-expressing tumours (VIII-IX *versus* II-III). Combined treatment with DBZ and bevacizumab enhanced necrosis in all groups (X-XII *versus* I-III).

### Blockade of DLL4 inhibited JAG1-tumour growth and increased vessel branching

**Figure 7:**
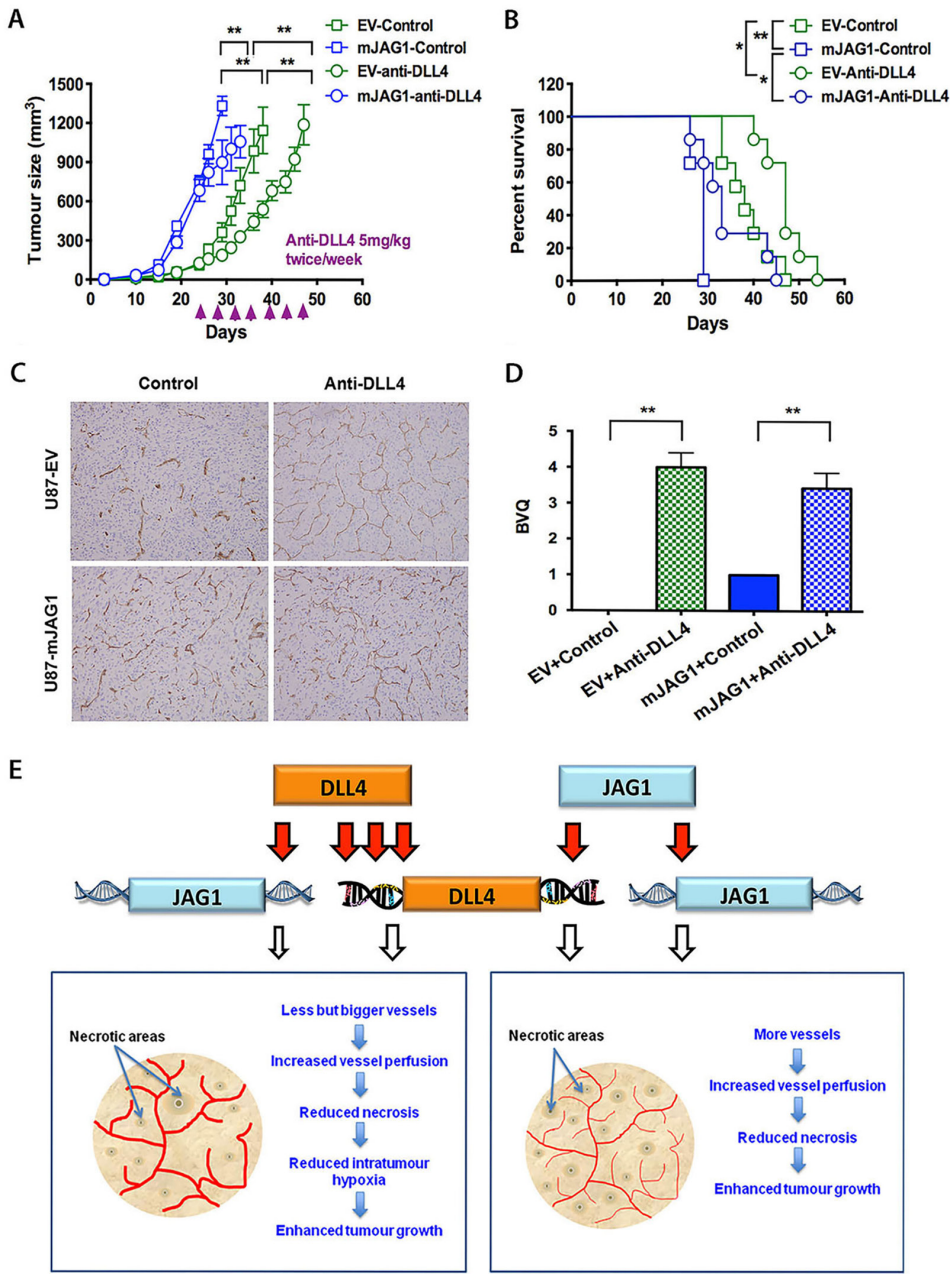
Anti-DLL4 antibody inhibited ***in vivo*** growth of U87-EV and U87-mJAG1 tumours and increased vessel branching. **A**. Tumour growth curves of U87-EV and U87-mJAG1 treated with anti-DLL4 blocking antibody (5mg/kg body weight) or PBS control. Each group consisted of 7 mice. Parametric generalized linear model with random effects was used for statistical test. **B**. Anti-DLL4 antibody increased tumour-bearing mouse survival. Kaplan-Meier analysis for overall survival test. Each group consisted of 7 mice. **C**. Visualisation of tumour xenograft vasculatures as revealed by CD34 staining. **D**. Quantification of vessel branching by branching vessel index (BVQ). Each group consisted of 7 tumours. Student's t-test. *P<0.05, ** *P*<0.01, *** *P*<0.001. **E**. A model demonstrating the effects of DLL4 or JAG1 on tumour angiogenesis and tumour growth. Angiogenic sprouting is aided by filopodia in response to VEGF gradient. DLL4-Notch signalling is promoted in vessels and tumours by increasing downstream gene expression. Signalling from tip cell to stalk cell specifies cell fate contributing to tumour growth. DLL4 and JAG1 amply Notch signalling by increasing their expressions. DLL4 is downstream of JAG1. JAG1 requires DLL4 for effect rather than antagonising it. The less induction of DLL4 by JAG1 than by DLL4 itself may produce less extreme phenotype with an increase in tip cells induced by JAG1 balanced by differentiation of stalk cells, implying a model of reciprocal regulation of the two cell populations. Both DLL4 and JAG1 promoted tumour growth but effectively reduced tumour necrosis through formation of different vasculature morphology.

To further investigate the interplay between JAG1 and DLL4 in promoting tumour growth, we used specific anti-DLL4 mAb (MedImm) that recognises both human DLL4 and mouse DLL4 to block DLL4-induced Notch signalling in mJAG1-expressing tumours. Blocking DLL4-Notch signalling with anti-DLL4 mAb significantly delayed tumour growth (Figure 7A) and increased mouse survival in both EV- and mJAG1-tumours (Figure 7B). Immunohistochemical staining for CD34 (Figure 7C) revealed that the treatment with anti-DLL4 mAb strikingly increased vessel branching in both EV- and mJAG1-tumours (Figure 7D), suggesting a predominant role of DLL4 in the control of tumour angiogenesis and tumour growth in either EV-tumours or mJAG1-tumours.

## DISCUSSION

mDLL4-induced Notch signalling appeared to be greater than mJAG1-induced signalling. mDLL4 increased endogenous DLL4 expression in a feed-forward manner [30] while mJAG1 increased DLL4 expression to a lesser extent. However, mDLL4 inhibited sprouting while mJAG1 induced sprouting angiogenesis. Such opposing effects were validated by overexpression and knockdown of endogenous ligands. Indeed, increased DLL4 signalling has been shown to synchronise fluctuations of DLL4 expression in individual ECs [25] and to change tip cells to stalk cells therefore reducing sprouting [27, 31] and driving vessel expansion [25]. JAG1 signalling was reported to be weak and antagonise DLL4 signalling, thus leading to an increase in tip cell formation and sprouting [26]. However, we showed that both DLL4- and JAG1-Notch signalling are significantly increased by either mDLL4 or mJAG1 *in vitro*.

mDLL4 and mJAG1 reduced cell proliferation *in vitro* but increased tumour growth *in vivo* as described for hDLL4 previously [7], suggesting a key role of tumour microenvironment. Indeed, both ligands reduced pericyte coverage. This reduction did not lead to a decrease in vessel stability. The vessels were in fact better perfused, suggesting that enhanced Notch signalling result in increased vessel stability either *via* the ECs themselves or *via* tumour-EC interactions. Recently it was reported that tumour cells can differentiate into ECs [32, 33] but we have found no such evidence in our xenograft models. Crosstalks between Notch and Wnt signalling induced by DLL4 [34] and JAG1 [35] have recently been shown to play a role in vessel stability. JAG1 has been shown to positively regulate vascular smooth muscle cell (SMC) coverage in certain models [26, 36, 37]; however Notch signalling has been shown to inhibit aortic SMC differentiation through an RBP-Jκ/Hey-dependent mechanism [38].

Tumour immunostaining revealed different vessel phenotypes. mDLL4 decreased vessel number but harboured bigger vessels as described previously with hDLL4 [6, 7] while JAG1 increased tumour vessel numbers only. Both types of vasculature were capable of increasing tumour growth *via* increased perfusion and decreased necrosis. Both ligands upregulated mHEY1 more than hHEY1, implying that paracrine signalling from tumour cells to stromal cells is stronger than autocrine signalling within tumour cells, supporting a vasculature-dependent mechanism for the enhanced tumour growth.

The effects of DLL4-Notch signalling on stromal cells in tumour microenvironment may be greater than that of mJAG1-Notch signalling. Indeed, endogenous DLL4 expression was higher in tumour vessels, particularly in those larger vessels which are known to have greater perfusion [7] and more resistant to anti-VEGF therapy [6]. Enhanced DLL4 expression by mDLL4 was also found in tumour and stromal cells. These stromal cells include tumour-associated macrophages [39, 40] which are known to accumulate in hypoxic areas in tumours, particularly in tumours treated with anti-VEGF therapy [41]. *In vitro* co-culture assays supported the findings *in vivo*. Both mDLL4 and mJAG1 expressed on different types of tumour cells strongly stimulated the expression of endogenous hDLL4 and hJAG1 in neighbouring cells including tumour cells themselves and HMEC-1 cells. Such autocrine and paracrine feed-forward signalling were much higher for induction of endogenous hDLL4 by mDLL4 than by mJAG1 in all types of tumour cells we have examined and in HMEC-1 ECs as well. Clearly, no antagonising effects between DLL4- and JAG1-Notch signalling were found.

Both mDLL4 and JAG1 xenografts had an increased sensitivity to DBZ compared to EV-tumours, suggesting that Notch signalling is responsible for the enhanced tumour growth [6]. We have previously demonstrated that hDLL4 is responsible for tumour resistance to anti-VEGF therapy [2, 6]. Here we found that mDLL4 and mJAG1 also conferred tumour resistance to bevacizumab in which the resistance triggered by mDLL4 was greater than that of mJAG1. Combination treatment had a synergistic efficacy against mJAG1-tumours as a complete reversal of the growth to even below the control level was achieved. It was reported that Notch signalling controls macrophage recruitment during angiogenesis and DLL4 on ECs activates Notch signalling on macrophages [42, 43]. This further enhancement by DLL4 may also contribute to its superior resistance.

The variation in vasculature phenotypes between mDLL4 and mJAG1 tumours may be explained by differences in downstream signalling activated by these ligands. DLL4 stimulation but not JAG1 was found to markedly induce EphrinB2 expression in ECs [44]. EphrinB2 is a key player in vessel size in angiogenesis [45] and arteriogenesis [46]. In fact, we found previously that EphB4-EphrinB2 signalling activated in DLL4-expressing tumours is, to some extent, responsible for the increased vessel size and tumour resistance to anti-VEGF therapy [6].

Expression of mJAG1 increased vessel numbers in xenograft tumours. Indeed, an increase in sprouting angiogenesis has been demonstrated in the retina of mice with JAG1 gain-of-function [26] and tumour models with such mice [28]. It was suggested that JAG1 is antagonising Notch signalling in the stalk cells to reverse the reduction in VEGFR2 expression induced by DLL4 signalling in the retina model [26] and in adult mouse skin wound healing [27]. However, this cannot explain the phenotypes we observed in our xenografts. Implantation of an equal amount of mDLL4- and mJAG1-expressing tumour cells in mice did not shown significant growth difference among mDLL4-tumour, mJAG1-tumour and mDLL4/mJAG1-tumour (see Figure 2C), suggesting no such antagonism for tumour growth. Perhaps, expression of DLL4 and/or JAG1 in different cell types such as ECs, tumour cells or other stromal cells might have different effects due to the lateral inhibition [25]. Notch signalling is significantly increased by JAG1 *in vitro* and *in vivo*, especially in tumour stromal cells. In addition both mDLL4- and mJAG1-tumours are resistant to bevacizumab treatment. If mJAG1 were reversing the level of VEGFR2 in tumour vessels a greater response to bevacizumab would be expected. However bevacizumab only partially reduced tumour growth and was capable of decreasing vessel density in both tumours.

To investigate this further, we treated the mJAG1-tumours with anti-DLL4 blocking antibody that recognises both human DLL4 and mouse DLL4. If mJAG1 were antagonising DLL4 signalling, anti-DLL4 treatment should have a little effect. However, we found that anti-DLL4 antibody was still capable of inhibiting tumour growth of both EV-tumour and mJAG1-tumour significantly. Blocking DLL4 signalling using anti-DLL4 antibody effectively delayed tumour growth possibly by disrupting functional angiogenesis [6–8, 19, 20, 47], implying that DLL4 is a dominant ligand over JAG1 in tumour angiogenesis and tumour growth.

In all xenograft models we tested, the same effects of mDLL4 and mJAG1 on the vascular phenotype were found, that is, mDLL4 reduced vessel numbers but increased vessel sizes while mJAG1 only increased vessel numbers. However, only in U87 xenograft model both mDLL4 and mJAG1 increased the tumour growth. In fact, such variability in tumour response to Notch-regulated angiogenesis has been already noted [7, 8, 19] and potentially related to many factors such as changes in cancer stem cell phenotype in the tumours [12], dependence on vascular co-option *versus* angiogenesis, and different microenvironments between different tumour types.

Altogether, we propose a model (Figure 7E) to depict the role of DLL4 and JAG1 in tumour angiogenesis and growth. JAG1 can enhance angiogenesis not by antagonising DLL4 but in a manner that requires DLL4 induction. JAG1 can induce DLL4 expression but to a lesser extent than DLL4 itself. DLL4 decreases vessel number but increases vessel size. Both DLL4 and JAG1 increase vessel perfusion and thus reduce necrosis and enhance tumour growth. Our results may have implications for development of anti-Notch therapies and suggest that anti-JAG1 therapy should be explored in conjunction with anti-DLL4 treatment in clinics.

## MATERIALS AND METHODS

### Cell culture and reagents

Immortalised human dermal microvascular endothelial cells (HMEC-1) were obtained from from ATCC through the CDC [48]. Human umbilical vein endothelial cells (HUVEC) were isolated from fresh umbilical cords [10]. Human cancer cell lines, U87GM (U87) glioblastoma cells, SAS head and neck cancer cells, PC3 and DU145 prostate cancer cells, were purchased from ATCC. Cell line authentication was carried out by STR analyses (LGC Standards). Cell lines over-expressing empty vector (EV), murine DLL4 (mDLL4) and murine JAG1 (mJAG1) were generated by retrovirus transductions as described previously [7]. Cultures of the tumour cells (U87, SAS and PC3, DU145) and HUVEC cells were described previously [7]. HMEC-1 cells were grown in MCDB-131 medium supplemented with 10% FCS, 100U/ml penicillin, 100*μ*g/ml streptomycin, 10mM L-glutamine, 10ng/ml epidermal growth factor (Sigma), and 1μg/ml hydrocortisone (BD Biosciences, Heidelberg, Germany). Recombinant human DLL4 (rhDLL4, Catalog no 1506-D4-050) and rat JAG1 (rrJAG1, Catalog no 599-JG-100) were purchased from R&D. Dibenzazepine (DBZ), a γ-secretase inhibitor, was purchased from Syncom (The Netherlands) at a purity of 99.8% at 232nm. Anti-DLL4 blocking mAb, a human IgG1 isotype that cross-reacts with both human DLL4 and mouse DLL4 (MedImm) was supplied as previously [49]. Anti-JAG1 mAbs, clone 65D that reacts with human JAG1 only with a high affinity and clone 142B that cross-reacts with human and mouse JAG1 with a low affinity compared to 65D, were made in-house.

### *In vitro* cell proliferation assay

Cells were seeded onto 6-well plates at 0.5-2 × 10^5^ cells per well (depending on cell lines) and incubated in the CO_2_ incubator at 37°C. Cells were counted everyday over 5-6 days using the Z2 Coulter Counter (Beckman Coulter).

### Real-time quantitative PCR (QPCR)

RNA extraction from tumour tissues and QPCR were performed as described previously [7]. Primers and probes for QPCR were designed using the Probe Library Assay Design Centre website (Roche Applied Science, Burgess Hill). Primer sequences and probes are: human GAPDH forward primer 5’-AGCCACATCGCTCAGACAC-3’, reverse primer 5’-GCCCAATACGACCAAATCC-3’ and probe 60; human ACT forward primer 5’-GAGGAGGCACCGGTAAATG-3’, reverse primer 5’-GTCACTCACTGGGACATAGGC-3’ and probe 81; human DLL4 forward primer 5’-CCCTGGCAATGTACTTGTGAT-3’, reverse primer 5’-TGGTGGGTGCAGTAGTTGAG-3’ and probe 23; human JAG1 forward primer 5’-GAATGGCAACAAAACTTGCAT-3’, reverse primer 5’-AGCCTTGTCGGCAAATAGC-3’ and probe 42; human HEY1 forward primer 5’-CGAGCTGGACGAGCCCAT-3’, reverse primer 5’-GGAACCTAGAGCCGAACTCA-3’ and probe 39; human HEY2 forward primer 5’-GTACCATCCAGCAGTGCATC-3’, reverse primer 5’-AGAGAATTCAGGGCATTT-3’ and probe 60; mouse ACT forward primer 5’-AAGGCCAACCGTGAAAAGAT-3’, reverse primer 5’-GTGGTACGACCAGAGGCATAC-3’ and probe 56; mouse HEY1 forward primer 5’-CATGAAGAGAGCTCACCC-3’, reverse primer 5’-CGCCGAACTCAAGTTTCC-3’ and probe 17. All primers were synthesised by Invitrogen, and probe Library was purchased from Roche Applied Science.

### Small interfering RNAs

The small interfering RNAs (siRNAs) were designed using the siDESIGN Center website (Dharmacon, Lafayette, USA) and synthesised by Eurogentec (Southampton). siRNA sequences are: scrambled control sense 3’-UCUGAAAAGCACGCUUGAC-5’ and antisense 3’-GUCAAGCGUGCUUUUCAGA-5’; siDLL4 Duplex1 sense 3’-CAACUAUGCUUGUGAAUGU-5’ and antisense 3’-ACAUUCACAAGCAUAGUUG-5’; siDLL4 Duplex2 sense 3’-ACACAAACCAGAAGAAGGA-5’ and antisense 3’-UCCUUCUUCUGGUUUGUGU-5’; siJAG1 Duplex1 sense 3’-AUCUUAUGAGGGAUUUACG-5’ and antisense 3’-CGUAAAUCCCUCAUAAGAU-5’; siJAG1 Duplex2 sense 3’-AACAGGACAAACAAACAGG-5’ and antisense 3’-CCUGUUUGUUUGUCCUGUU-5’. HMEC-1 cells were transfected with siRNA (20nM for siDLL4 and 5nM for siJAG1) using HiPerfect (QIAGEN) according to manufacturer's instructions.

### Hanging drop assay

HMEC-1 cells were suspended in medium containing 0.2% (w/v) carboxymethylcellulose and seeded in non-adherent 60-microwell minitrays (Sigma). Spheroids with defined size and cell number (500 cells/spheroid) were generated overnight. They were then embedded into Matrigel in a 24-well plate and allowed to polymerize before adding medium. Pictures were taken at 10× magnifications using an Axiovert 100M microscope (Zeiss, Germany). Capillary sprouting was assessed by measuring the average length of three longest sprouts per spheroid [50] originating from 15 randomly selected spheroids per well on day 3 when sprouting was most eminent, using Image J.

### Flow cytometry

Cell co-culture of retrovirus transduced tumour cells or EC cells, which are expressing GFP protein encoded by the retrovirus vector, with an equal amount of each parental tumour or EC line (including U87, SAS, PC3, DU145 or HMEC-1) (50:50 ratio) were performed in 6-well plates or 10-cm cell culture dishes in the CO_2_ incubator at 37°C for 2-3 days until the confluence of cell growth. The cells were then harvested for FACS staining by incubating with a primary antibody at 4°C for 30 minutes. Primary antibodies used include MedImm human anti-DLL4 blocking mAb (which cross-reacts with both human DLL4 and mouse DLL4) and in-house made mouse anti-JAG1 mAbs (clone 65D reacts with human JAG1 only with a high affinity while clone 142B cross-reacts with human JAG1 and mouse JAG1 with a low affinity compared to 65D). After washed with PBS for 3 times each 3-5 minutes, the cells were incubated with PE- or APC-conjugated secondary antibodies at 4°C for 30 minutes. After washed with PBS, the cells were sorted for GFP-negative and GFP-positive portions on the BD Fortessa X-20 FACS machine and analysed for the expression of DLL4 and JAG1 with the FlowJo software.

### Xenograft mouse models

Animal experiments were performed as previously described [7] under Home Office legislation. DBZ (8.1 µmol/kg of body weight) and bevacizumab (10mg/kg of body weight) were given intraperitoneally every three days, starting when the tumour reached 150mm^3^ in size [6]. MedImm anti-DLL4 blocking mAb (5 mg/kg body weight) was administered twice a week starting when EV-control tumour reached 150mm^3^ in size. Mice were sacrificed when reach to the criteria defined in the animal project license.

### Immunostaining

The mouse CD31 and NG2 staining protocol on frozen sections and quantification were performed as described previously [7]. For the xenografts treated with anti-DLL4 antibody A visual vessel scoring method was employed to determine the number of branched vessels (BVQ). A score of 0-5 was given by two independent observers for vessel scoring. Biotinylated Lycopersicon Esculentum (tomato) lectin (1µg/µl) was injected intravenously into tail vein 10 min prior to euthanasia to determine vessel perfusion [7]. Images were taken from the most vascularised areas to determine percentage of perfusion in existing vessels. Percentage of perfusion was calculated as the percentage area positive for tomato lectin compared to the CD31 positive area. DLL4 and JAG1 expression was detected with anti-DLL4 antibody (1:100, Abcam) and anti-JAG1 antibody 28H8 (1:100, Cell Signalling). For immunofluorescent staining, Alexa-Fluor 546 donkey anti-goat (0.5 μg/mL, Molecular Probe, Invitrogen) and Alexa-Fluor 488 donkey anti-rabbit (0.5 μg/mL, Molecular Probe, Invitrogen) secondary antibodies were used. Samples were sequentially scanned with a Zeiss LSM 510 Meta 63X objective (Plan-achromat 63X 11.40 oil immersion). CAIX staining was detected using CAIX M75 antibody at 1:50 as described previously [7]. Necrosis was quantified histologically on hematoxylin-stained sections [6].

### Statistical analysis

Prism software was used to analyse the data. The nalysis of variance (ANOVA) test was used to compare mean values among three or more data sets, and the Bonferroni's post-test to compare any two data sets among the three or more sets. Non-parametric Kruskal-Wallis test with Dunn's post-test comparison was also employed. A generalized linear model with random effects for tumour growth was used to analysis growth curves. ANOVA F-test was used to assess significance between curve fits. Kaplan-Meier survival curves were used to analyse the percentage survival of mice. All data were presented as Mean±SEM except when indicated specifically in figure legends. Statistical significance was indicated in figures with an asterisk where *P* < 0.05, two asterisks where *P* < 0.01 and three asterisks where *P* < 0.001.

## SUPPLEMENTARY MATERIALS FIGURES


