# Supplementary material for: Role of Delta-like 4 in Jagged1-induced tumour angiogenesis and tumour growth
Source: Oncotarget. 2017 Apr 8;8(25):40115–31. doi: 10.18632/oncotarget.16969 (PMC5522274; doi:10.18632/oncotarget.16969)
Supplement: Supplementary file 1 [file oncotarget-08-40115-s001.pdf]

# Role of Delta-like 4 in Jagged1-induced tumour angiogenesis and tumour growth

## Supplementary Material

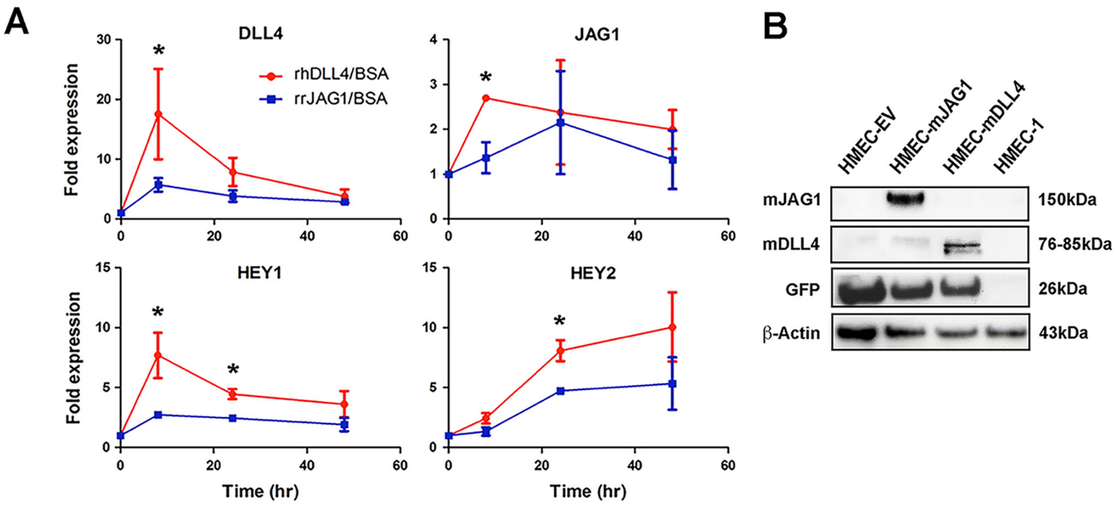

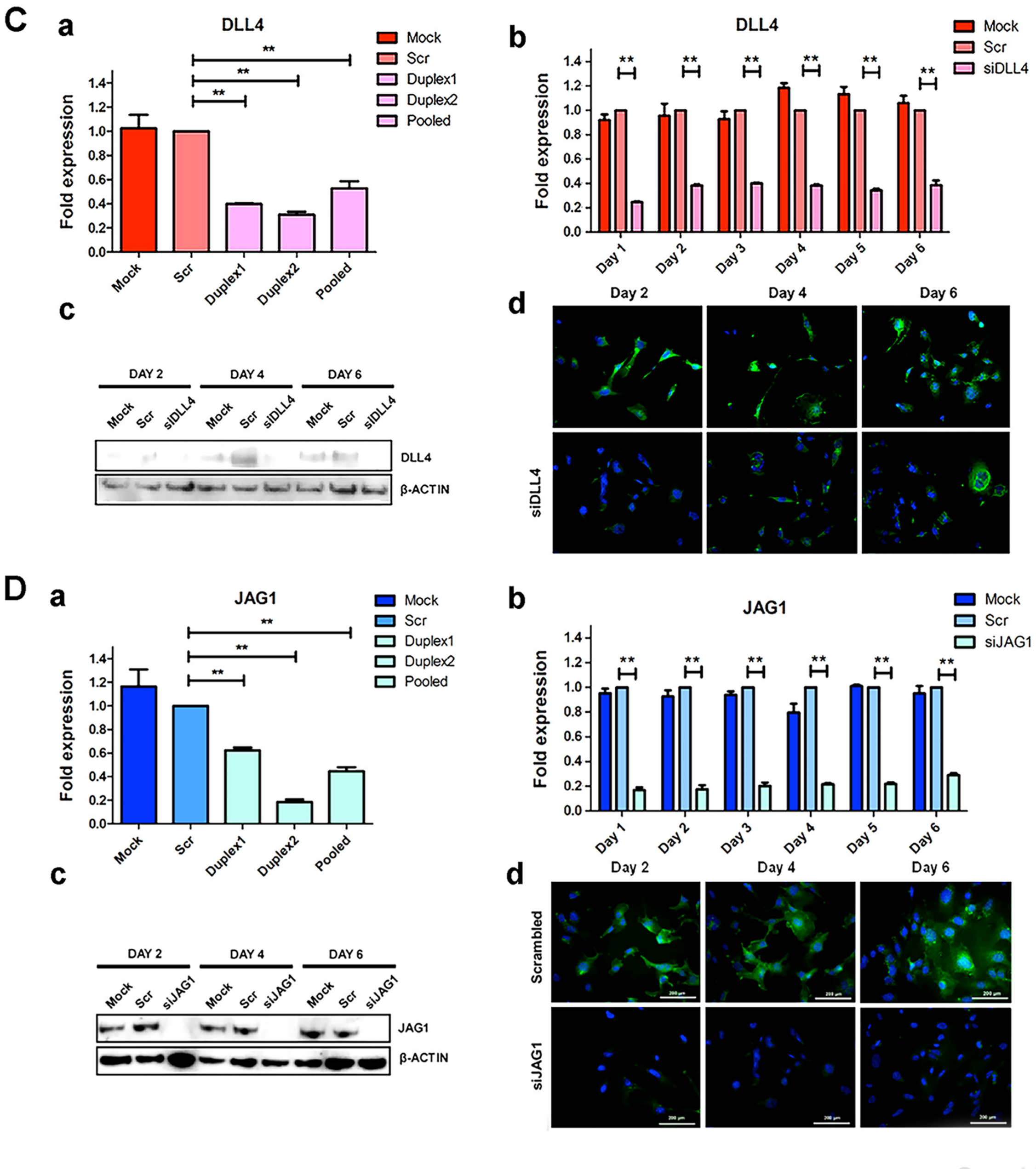


**Supplementary Figure 1.** DLL4 and JAG1 activated Notch signalling and affected sprouting angiogenesis in vitro.

**A) DLL4 activated Notch signalling stronger than JAG1 in primary HUVEC cells.** Expression profile of Notch target genes in HUVEC cells stimulated with 18nM rhDLL4 or rrJAG1 coated on the plate over a time course. QPCR was used to determine mRNA levels of DLL4, JAG1, HEY1 and HEY2. Fold changes were obtained by normalizing against the EV control. Student's t-test.

**B) Upregulation of mDLL4 and mJAG1 in HMEC-1 cells by retrovirus transductions.** Western blotting confirmed the expression of mDLL4 and mJAG1. GFP encoded by the retrovirus vector was served as an internal control for the retrovirus transduction efficacy. β-actin served as a protein loading control.

**C) Knockdown of endogenous hDLL4 in HMEC-1 cells by siRNAs.** QPCR confirmed the knockdown of hDLL4 expression at the mRNA level (a) by two different siRNA duplexes (20nM), compared to scrambled (Scr) control. Time course expression of hDLL4 at the mRNA level (b) and at the protein level confirmed by Western blotting (c) and immunofluorescence staining (d). Representative figures were shown (n=2 independent experiments). ANOVA with Bonferroni's post-test.

**D) Knockdown of endogenous hJAG1 in HMEC-1 cells by siRNAs.** QPCR confirmed the knockdown of hJAG1 expression at the mRNA level (a) by two different siRNA duplexes (20nM), compared to scrambled (Scr) control. Time course expression of hJAG1 at the mRNA level (b) and at the protein level confirmed by Western blotting (c) and immunofluorescence staining (d). Representative figures were shown (n=2 independent experiments). ANOVA with Bonferroni's post-test.

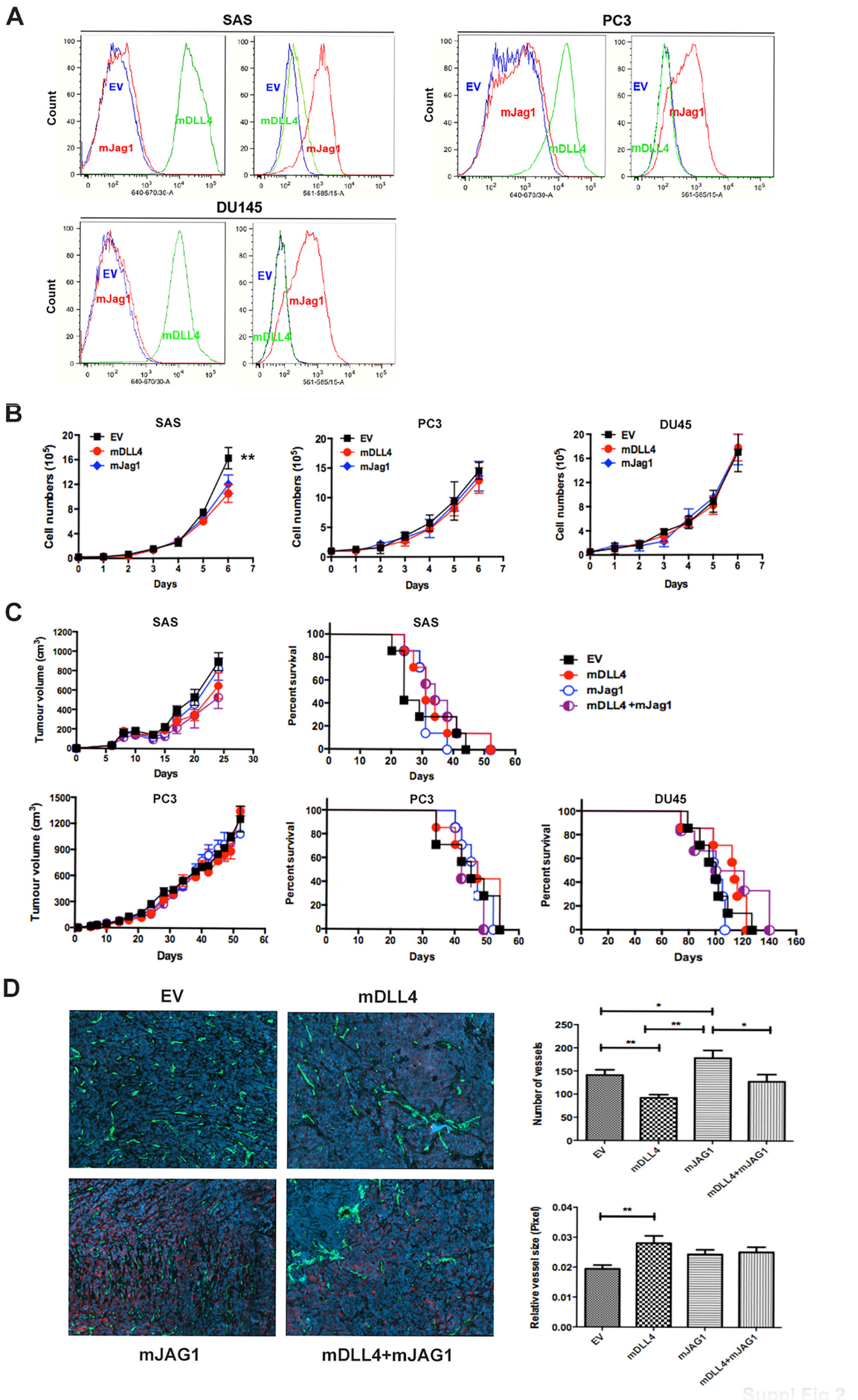


**Supplementary Figure 2.** Effects of mDLL4 and mJAG1 on cell growth in vitro and tumour growth in vivo of three tumour cell lines and on their tumour vasculature

**A) FACS staining confirmed the over-expression of mDLL4 and mJAG1 in SAS, PC3 and DU145 tumour cells compared to its EV control, respectively.**

**B) Effects of mDLL4 and mJAG1 expressed in tumour cells on cell growth** *in vitro.* Representative figures were shown (3 independent experiments). Error bars represent SD.

**C) Effects of mDLL4 and mJAG1 expressed in tumour cells on xengoraft growth or mouse overall survival** *in vivo.*

**D) Effects of mDLL4 and mJAG1 expressed in tumour cells on their vascular phenotypes.** Immunofluorescence triple staining for cell nuclei with DAPI (blue), tumour vessels with anti-CD31 antibody (green) and JAG1 expression with anti-JAG1 antibody (red) and quantifications of vessel number and vessel size were performed on tumour sections of SAS cells. Each group consisted of 7 tumours. ANOVA with Bonferroni's post-test.

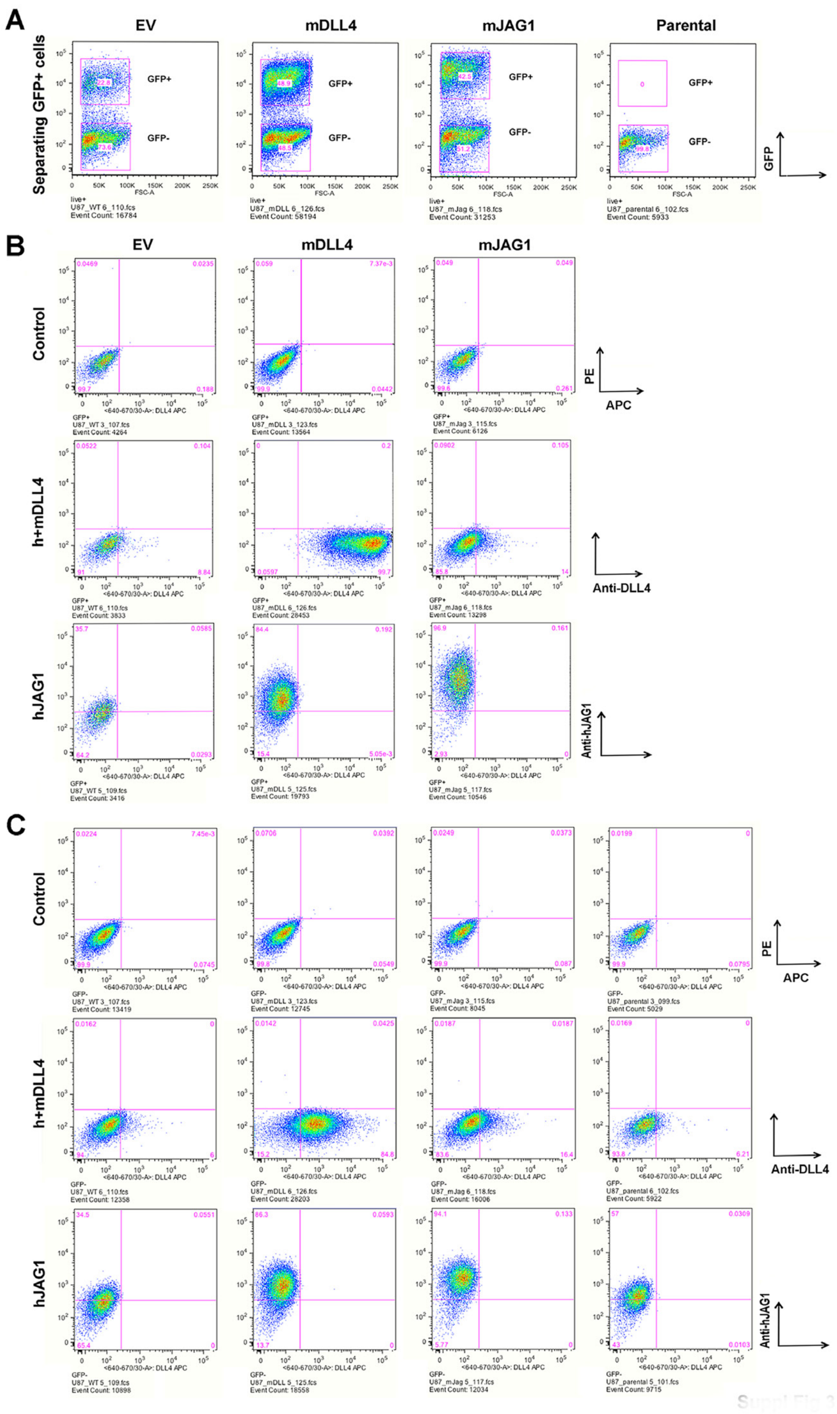


**Supplementary Figure 3.** FACS analysis strategy for detection of endogenous hDLL4 and hJAG1 expression in four tumour cell lines and HMEC-1 cells.

**A) FACS analysis for detection of endogenous ligand expression.** Co-culture of parental U87, U87-EV, U87-mDLL4, U87-mJAG1 or mixture of U87-mDLL4 and U87-mJAG1 cells (containing GFP encoded by the retrovirus vector) with an equal numbers of parental U87 cells (GFP-negative) (50:50 ratio) in 6-well plates for 3 days, FACS staining for DLL4 and JAG1 with specific antibodies, and then separation of GFP-positive and GFP-negative cells on the BD Fortessa X-20 FACS machine. The expression of DLL4 or JAG1 in GFP-positive cells and GFP-negative cells were then analysed with the FlowJo software.

**B) FACS analysis for the expression of DLL4 or JAG1 in GFP-positive U87 cells with MedImm human anti-DLL4 blocking mAb that cross-reacts with both human and mouse DLL4 and with 65D mouse anti-human JAG1 mAb.**

**C) FACS analysis for the expression of DLL4 or JAG1 in GFP-negative U87 cells with MedImm human anti-DLL4 blocking mAb and with 65D mouse anti-human JAG1 mAb.**

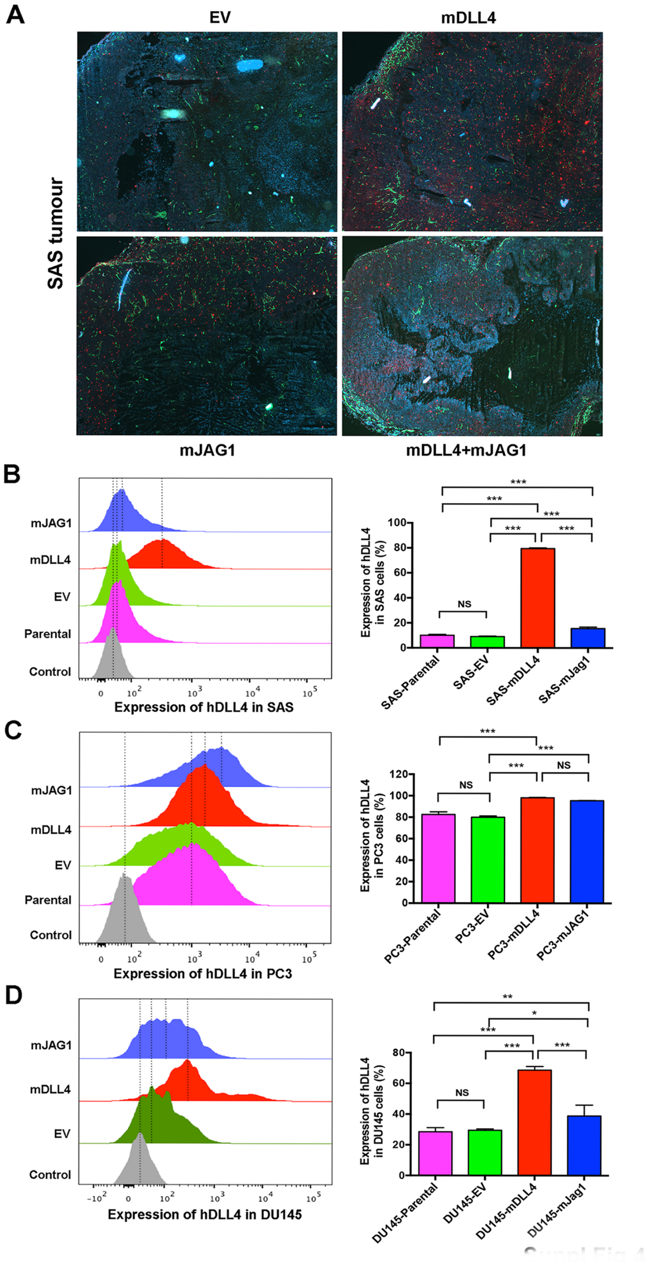


**Supplementary Figure 4.** DLL4 and JAG1 induced endogenous DLL4 expression in tumour tissues and tumour cells

**A) Immunofluorescence triple staining for cell nuclei with DAPI (blue), tumour vessels with anti-CD31 antibody (green) and DLL4 expression with Santa Cruz anti-DLL4 antibody C-20 (red) on tumour sections of SAS-EV, SAS-mDLL4, SAS-mJAG1, and mDLL4+mJAG1-tumours.**

**B) Endogenous hDLL4 expression in parental SAS cells (GFP-negative) sorted from co-culture of parental SAS, SAS-EV, SAS-mDLL4 or SAS-mJAG1 (GFP-positive) with an equal amount of parental SAS cells detected by FACS staining with MedImm anti-DLL4 blocking mAb (that recognises both human DLL4 and mouse DLL4).** ANOVA with Bonferroni's post-test. N=3. NS, no statistical difference. Error bars represent SD.

**C) Endogenous hDLL4 expression in parental PC3 cells (GFP-negative) sorted from co-culture of parental PC3, PC3-EV, PC3-mDLL4 or PC3-mJAG1 (GFP-positive) with an equal amount of parental PC3 cells detected by FACS staining with MedImm anti-DLL4 blocking mAb (that recognises both human DLL4 and mouse DLL4).** ANOVA with Bonferroni's post-test. N=3. NS, no statistical difference. Error bars represent SD.

**D) Endogenous hDLL4 expression in parental DU145 cells (GFP-negative) sorted from co-culture of parental DU145, DU145-EV, DU145-mDLL4 or DU145-mJAG1 (GFP-positive) with an equal amount of parental DU145 cells detected by FACS staining with MedImm anti-DLL4 blocking mAb (that recognises both human DLL4 and mouse DLL4).** ANOVA with Bonferroni's post-test. N=3. NS, no statistical difference. Error bars represent SD.

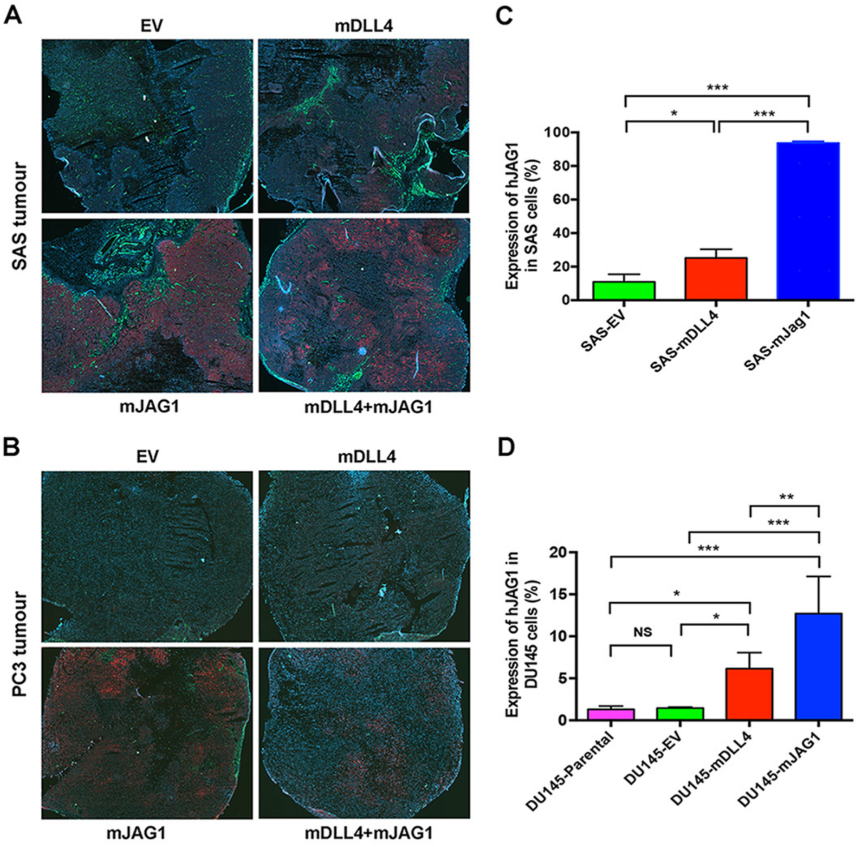


**Supplementary Figure 5.** DLL4 and JAG1 induced endogenous JAG1 expression in tumour tissues and tumour cells

**A) Immunofluorescence triple staining for cell nuclei with DAPI (blue), tumour vessels with anti-CD31 antibody (green) and JAG1 expression with Cell Signalling anti-JAG1 antibody 28H8 (red) on tumour sections of SAS-EV, SAS-mDLL4, SAS-mJAG1 and SAS-mDLL4+mJAG1 tumours.**

**B) Immunofluorescence triple staining for cell nuclei with DAPI (blue), tumour vessels with anti-CD31 antibody (green) and JAG1 expression with Cell Signalling anti-JAG1 antibody 28H8 (red) on tumour sections of PC3-EV, PC3-mDLL4, PC3-mJAG1 and PC3-mDLL4+mJAG1 tumours.**

**C) Endogenous hJAG1 expression in SAS cells (GFP-positive) sorted from co-culture of SAS-EV, SAS-mDLL4 or SAS-mJAG1 (GFP-positive) with an equal amount of parental SAS cells (GFP-negative) detected by FACS staining with 65D anti-hJAG1 mAb.** ANOVA with Bonferroni's post-test. N=3. Error bars represent SD.

**D) Endogenous hJAG1 expression in parental DU145 cells (GFP-negative) sorted from co-culture of parental DU145, DU145-EV, DU145-mDLL4 or DU145-mJAG1 (GFP-positive) with an equal amount of parental DU145 cells detected by FACS staining with 65D anti-hJAG1 mAb.** ANOVA with Bonferroni's post-test. N=3. NS, no statistical difference. Error bars represent SD.
